# The Characteristics of Severely Injured Trauma Patients Admitted to a Level I Trauma Center with Pre-Injury Use of Oral Anticoagulation (OAC) or Antiplatelet Therapy (APT)

**DOI:** 10.3390/jcm14103614

**Published:** 2025-05-21

**Authors:** Valerie Weihs, Anna Antoni, Stephan Frenzel, Silke Aldrian, Stefan Hajdu, Lukas L. Negrin

**Affiliations:** Department of Orthopaedics and Trauma Surgery, Division for Trauma Surgery, Medical University of Vienna, 1090 Vienna, Austria; anna.antoni@meduniwien.ac.at (A.A.); stephan.frenzel@meduniwien.ac.at (S.F.); silke.aldrian@meduniwien.ac.at (S.A.); stefan.hajdu@meduniwien.ac.at (S.H.); lukas.negrin@meduniwien.ac.at (L.L.N.)

**Keywords:** oral anticoagulation, antiplatelet therapy, severely injured, trauma patients

## Abstract

**Background**: Little is known about the impact of pre-injury OAC/APT on severely injured trauma patients admitted to a level I trauma center. Our study focused on impact of pre-injury OAC/APT on the outcomes of this specific cohort of patients. **Methods**: A retrospective cohort study on 356 severely injured trauma patients admitted to the resuscitation room in a level I trauma center between 2015 and 2020 was carried out. **Results**: Of the 356 patients, 20.5% (n = 73) were admitted with pre-injury OAC/APT. Falls from lower heights, categorized as low-energy trauma, were the primary mechanism of injury in patients with pre-injury OAC/APT. Patients with pre-injury OAC/APT were older (*p* < 0.001), had a higher proportion of severe traumatic brain injuries (TBI) (*p* < 0.001), and experienced significantly higher mortality rates during their hospital stay (60.3% vs. 15.9%; *p* < 0.001). There were significant correlations between pre-injury OAC/APT and advanced age (*p* < 0.001) as well as the severity of head injury (*p* < 0.001). Patients with pre-injury OAC/APT exhibited significantly higher mortality rates; especially in patients with pre-injury oral anticoagulation therapy. The highest mortality rates were observed in patients with isolated TBI and pre-injury OAC/APT. **Conclusions**: Trauma patients with pre-injury OAC/APT presented with advanced age and low-energy trauma as the main mechanism of injury. Pre-injury OAC/APT significantly correlated with advanced age and the severity of head injuries, and it may serve as an additional prognostic factor for the outcome of severely injured trauma patients.

## 1. Introduction

Over the past few decades, an increasing number of geriatric trauma patients have been documented [1,2,3]. Globally, the burden of cardiovascular diseases is consistent with the number of patients with oral anticoagulation (OAC) or antiplatelet therapy (APT), which is continuously increasing [4]. Little is known about the impact of pre-injury OAC or APT on the outcome of the severely injured patient arriving at the resuscitation room [5,6,7], despite the fact that injury-related factors such as the injury mechanism, older age, pre-existing comorbidities, and pre-injury medications were found to influence the outcome of polytrauma patients [8,9,10,11]. Overall, severely injured elderly patients seem to have higher mortality rates despite low-energy trauma mechanisms as the leading cause of injury [12]. Concomitant TBI seems to remain the predominant factor for the outcome of trauma patients [13,14]. When comparing trauma patients arriving in the resuscitation room, those with isolated traumatic brain injury (TBI) are significantly older and undergo considerably more neurosurgical interventions. Yet, there is no difference in mortality rates when compared to polytrauma patients with TBI [13]. The prognosis and prediction of mortality in trauma patients are essential factors in trauma care and depend on various aspects [15]. The hypothesis of our study was that severely injured trauma patients with pre-injury OAC or APT have worse outcomes with regard to mortality than severely injured trauma patients without pre-injury OAC or APT. We aimed to analyze the characteristics and the outcomes of severely injured trauma patients with pre-injury OAC or APT triaged to the resuscitation room and tried to answer the following questions:

- Are there any differences regarding the injury pattern or the clinical characteristics in trauma patients with pre-injury OAC/APT?
- Is the presence of pre-injury OAC/APT linked to the severity of TBI?
- Does pre-injury use of oral anticoagulants (OAC) or antiplatelet therapy (APT) affect the mortality rate of severely injured trauma patients admitted to the resuscitation room of a level I trauma center?

## 2. Materials and Methods

**Figure 1:**
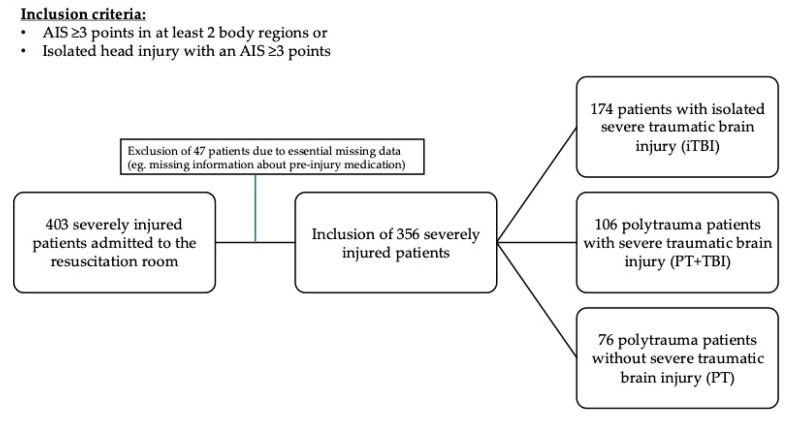
Inclusion and classification of severely injured patients. AIS: abbreviated injury scale; iTBI: isolated traumatic brain injury; PT: polytrauma; TBI: traumatic brain injury.

A retrospective study of adult, severely injured patients admitted to the resuscitation room in a level I trauma center was performed. Polytrauma (PT) patients with severe, multiple injuries (AIS ≥ 3 points in at least two body regions) and patients with isolated severe traumatic brain injury (iTBI) (head injury with an Abbreviated Injury Scale (AIS) ≥ 3 points) were enrolled starting from January 2015 to December 2020. Exclusion criteria were isolated severe injuries in any other body region, patients with minor injuries (ISS < 18 points), and patients < 18 years of age. Four hundred and three consecutive patients were included retrospectively. Of them, 47 patients needed to be excluded due to missing essential data (e.g., missing information about the pre-injury medication), leading to the remaining 356 patients (Figure 1).

The following demographic details were analyzed: sex, age, injury severity score (ISS), AIS, injury mechanism, pre-existing comorbidities, in-hospital complications, length of stay (LOS), and in-hospital mortality. Data were extracted from a consecutive in-hospital database of severely injured patients. The data records of all included patients were screened for pre-existing comorbidities and pre-injury OAC or APT. The primary outcome of interest was the influence of pre-injury OAC/APT on the survival rates during the hospital stay. Subanalyses were performed with regard to the different types of pre-injury medication (OAC or APT).

### Statistical Analysis

Continuous variables are presented as means and standard deviation or medians and interquartile range (IQR) depending on their normal or skewed distribution. The Kolmogorov–Smirnov test was used to assess the normal distribution. The Mann–Whitney U or Student’s *t*-test was performed to compare categorical and continuous variables, and the chi-square test was used to compare qualitative variables. The contingency coefficient was used to detect a potential correlation between categorical variables, and the Spearman correlation coefficient was used for detecting a potential correlation between categorical and continuous variables. To assess potential multicollinearity of age, TBI, and pre-injury OAC/APT; the variance inflation factor (VIF) was calculated. The Kaplan–Meier method with a log-rank test was used for mortality analysis. A univariate Cox regression analysis was performed to detect a potential prognostic factor for survival. Only significant factors (*p* < 0.05) from the univariate analysis were included in the multivariate Cox regression analysis. All statistical analyses were performed using IBM Statistics Version 29.0 (SPSS Inc. Chicago, IL, USA).

## 3. Results

A total of 356 patients were enrolled consecutively, with half of them being trauma patients with isolated severe TBI (iTBI; n = 174; 48.9%) and half of them being polytrauma patients (n = 182; 51.1%) presenting either as polytrauma patients with severe head injury (PT+TBI; n = 106) or as polytrauma patients without severe head injury (PT; n = 76). Most patients were male (n = 245; 68.8%) with a median age of 51 years (IQR 39) and a median ISS of 29 points (IQR 11). One hundred and fifteen patients (32.3%) were defined as geriatric (>64 years of age). About 1/5 of all patients were admitted with active pre-injury OAC or APT (n = 73; 20.5%), with equal parts of them presenting with any APT (n = 37; 50.7%) or any OAC (n = 36; 49.3%). Most patients had pre-injury single APT (n = 31; 8.7%), followed by direct OAC (n = 16; 4.5%) and vitamin-K-dependent OAC (n = 15; 4.2%). Dual APT was recorded in six patients (1.7%), a combination of APT and OAC in three patients (0.8%) and two patients had other antithrombotic therapy (low molecular weight heparin (LMWH)). Pre-existing comorbidities were documented in 60.7% of patients (n = 216). The primary mechanism of injury was any traffic-related accident (TRA) in 35.7% of cases (n = 127), followed by falls from lesser height in 31.2% of cases (n = 111), falls from greater height (n = 50; 14.0%), penetrating injuries (n = 19; 5.3%) and other/unknown causes of injury (n = 49; 13.8%). A total of 89 patients (25.0%) died during the hospital stay, with 36 of them during the first 24 h and 53 of them after 24 h during the hospital stay.

### Influence of OAC or APT

The characteristics of patients with OAC or APT are shown in Table 1.

**Figure 2:**
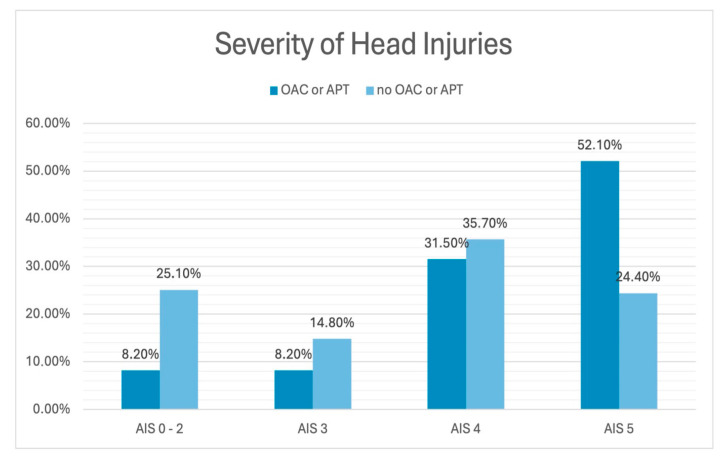
Frequency of the severity of head injuries with and without pre-injury OAC/APT. OAC: oral anticoagulation; APT: antiplatelet therapy; AIS: abbreviated injury scale.

The characteristics of severely injured patients with regard to pre-injury OAC/APT are listed in Table 1. Higher proportions of severe head injuries were documented in patients with pre-injury OAC/APT (*p* < 0.001) (Figure 2). Significant correlations of pre-injury OAC/APT with advanced age (*p* < 0.001; contingency coefficient 0.515) and the severity of head injury (*p* < 0.001; contingency coefficient 0.257) were seen. Age showed a significant correlation with a severe TBI (*p* < 0.001; Spearman correlation coefficient 0.573). The risk of a severe TBI showed an increase of 2% per year of aging (HR 1.020 95% CI 1.008–1.033 *p* = 0.002).

**Figure 3:**
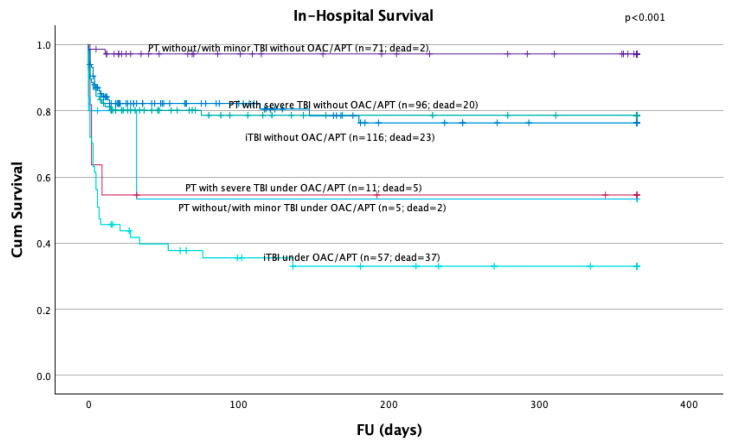
Survival of all patients with significantly higher mortality rates in patients with isolated severe TBI followed by polytrauma patients with or without head injuries with pre-injury OAC/APT. OAC: oral anticoagulation; APT: antiplatelet therapy; PT: polytrauma; TBI: traumatic brain injury; iTBI: isolated traumatic brain injury.

Kaplan–Meier analyses demonstrated the lowest survival rates in iTBI patients with pre-injury OAC/APT as well as in PT patients (with and without severe TBI) and pre-injury OAC/APT. In contrast, PT patients without pre-injury OAC/APT showed significantly higher survival rates (*p* < 0.001) (Figure 3). Patients with pre-injury OAC/APT tended to die more often after survival of the first 24 h (26 out of 44 in-hospital deaths; 59.1%). When compared to patients with APT, patients with pre-injury OAC therapy showed significantly higher mortality rates during the hospital stay (73.5% vs. 45.9%; *p* = 0.018) as well as after 24 h (50.0% vs. 24.3%; *p* = 0.025).

Univariate Cox regression analysis revealed advanced age > 64 years, severe TBI, pre-existing comorbidities, and pre-injury OAC/APT as potential prognostic factors for survival. Multivariate analysis identified severe TBI and pre-injury OAC/APT as independent prognostic factors for the survival of severely injured trauma patients (Table 2).

## 4. Discussion

Although a significant decline in injury-related deaths has been seen in the 1990s [16,17], the mortality rates have remained relatively constant over the past decades [3]. Those rates might be related to the significant increase in the mean age of trauma patients and the known influence of advanced age on their survival [2,16,18]. Accordingly, a significant increase in comorbidities in trauma patients, especially in the elderly ones, has been observed with an expected increase in pre-injury OAC or APT [10,11]. Most patients who arrive at the resuscitation room present either as PT victims with/without severe TBI or as patients with isolated severe TBI, with little difference in the mortality rates among the three groups [13]. In this study, we aimed to see whether pre-injury use of OAC/APT influences the outcome (defined as in-hospital mortality) on these three groups.

Initially, we observed a significant proportion of severe trauma patients with pre-injury OAC/APT who were admitted to the resuscitation room (one-fifth of all trauma patients). This specific group of patients showed some distinctive clinical characteristics: they presented with a significantly higher median age and showed distinctive injury mechanisms reflecting the advanced age and potential frailty. Most trauma patients with pre-injury OAC/APT were admitted after falls from lesser height as a low-energy injury pattern. In contrast to that, trauma patients without pre-injury OAC/APT were mainly admitted after traffic-related accidents. Despite known injury-related predictors such as type and site of injury, as well as injury severity [11,19,20,21,22,23,24]; advanced age, pre-existing comorbidities, and pre-injury medications were found to have a potential influence on the outcome of polytrauma patients previously [9].

The rates of head injuries increased, particularly among the elderly group of trauma patients [25]. As severe TBI and advanced age are significant prognostic factors for the outcomes of severely injured trauma patients (27), we sought to explore a potential correlation between advanced age, the presence of severe TBI, and pre-injury OAC/APT. We demonstrated that patients with pre-injury OAC/APT appeared significantly more frequently in iTBI patients. Additionally, pre-injury OAC/APT was significantly linked to the severity of head injuries and with advanced age. Age itself showed a significant correlation with the occurrence of severe TBI. Overall, there appears to be a strong relationship between advanced age, OAC/APT intake, and the incidence of severe head injuries in trauma patients.

With regard to the outcome of our patients with pre-injury OAC/APT, those with pre-injury OAC/APT showed the highest mortality rates irrespective of the type of trauma (iTBI or PT). Despite the significant higher mortality rate of patients with pre-injury OAC/APT, the injury severity was similar in both groups. This finding indicates the diminished ability of the ISS to underline singular injuries with major influence on the outcome such as severe head injuries. After multivariate analyses, severe TBI and pre-injury OAC/APT remained independent prognostic factors on the survival of our trauma patients. We demonstrated that with advanced age, the risk of experiencing a severe head injury increases significantly (2% per year of aging). Both severe TBI and pre-injury OAC/APT were closely associated with advanced age, showing a significant correlation between pre-injury OAC/APT and both advanced age and the severity of the head injury. An early detection of potential pre-injury OAC/APT in the elderly trauma patient might be beneficial and potentially should be implemented in specific trauma protocols for the elderly. This might lead to an early initiation of reversal agents and might reduce the severity of head injuries in the elderly leading to a better outcome in this specific cohort of patients.

Overall, the combination of concomitant TBI, advanced age, and pre-injury OAC/APT appears to be the primary factor influencing the outcomes of trauma patients transferred to the resuscitation room.

This study has the characteristic limitations of retrospective, single-center registry data and post hoc analyses with a relatively small sample size. No data on functional outcome or quality-of-life parameters were available. Despite careful data analysis and consecutive inclusion of patients, there might be an inherent selection bias. No data regarding the adherence of OAC/APT, the dosage of the medications or the activity of the drugs were available. Further studies focusing on severely injured patients on OAC/APT are needed with the implementation of prospective trials and specific protocols. Still, little is known about the clinical impact of point of care blood tests and potential reversal of pre-injury OAC/APT in severely injured patients.

## 5. Conclusions

Severely injured trauma patients with pre-injury OAC/APT display distinctive clinical characteristics, showing advanced age and low-energy trauma as the primary mechanisms of injury. Pre-injury OAC/APT closely correlates with advanced age and the severity of head injuries. Alongside severe TBI, pre-injury OAC/APT appears to be an independent predictor of survival for severely injured trauma patients.

## Figures and Tables

**Table 1:** Characteristics of severely injured patients. PT: polytrauma; TBI: traumatic brain injury; ISS: injury severity score; IQR: interquartile range. ° chi-square test. * Mann–Whitney U test.

	OAC or APT (n = 73)	no OAC or APT (n = 283)	*p*-Value
Female n (%)	18 (24.7)	93 (32.9)	0.177 °
Age median (IQR)	79 (13)	46 (32)	<0.001 *
PT without/with minor TBI (PT)	5 (6.8)	71 (25.1)	<0.001 °
PT with severe TBI (PT+TBI)	11 (15.1)	95 (33.6)	
Isolated TBI (iTBI)	57 (78.1)	117 (41.3)	
ISS median (IQR)	29 (14)	29 (12)	0.543 *
In-hospital death n (%)	44 (60.3%)	45 (15.9)	<0.001 °
Injury pattern			
Fall from lesser height n (%)	47 (64.4)	64 (22.6)	<0.001 °
Fall from greater height n (%)	3 (4.1)	47 (16.6)	
Traffic-related accident n (%)	12 (16.4)	115 (40.6)	
Penetrating n (%)	1 (1.4)	18 (6.4)	
Other/unknown n (%)	10 (13.7)	39 (13.8)	
In-hospital complications n (%)	66 (23.3)	18 (24.7)	0.811 °
Comorbidities	73 (100)	143 (50.5)	<0.001 °
Geriatric patients	64 (87.7)	51 (18.8)	<0.001 °

**Table 2:** Uni- and Multivariate Cox Regression Analysis on potential prognostic factors for the survival of severely injured trauma patients. OAC: oral anticoagulation; APT: anitplatelet therapy; TBI traumatic brain injury; HR hazard ratio; CI confidence interval.

	Univariate Cox Regression			Multivariate Cox Regression		
	*p*-Value	HR	95% CI	*p*-Value	HR	95% CI
Age > 64 years	<0.001	3.746	2.450–5.726	0.060	1.716	0.978–3.012
Comorbidities	<0.001	2.794	1.665–4.687	0.643	1.160	0.620–2.171
Pre-injury OAC/APT	<0.001	4.924	3.243–7.476	<0.001	2.806	1.624–4.851
Severe TBI	<0.001	5.436	2.204–13.410	0.005	3.672	1.470–9.169

## Data Availability

The datasets generated and analyzed in the current manuscript are not publicly available due to data protection regulations. The data used and analyzed in this study are available from the corresponding author solely upon reasonable request.

## Abbreviations

The following abbreviations are used in this manuscript:

OAC	Oral anticoagulation
APT	Antiplatelet therapy
TBI	Traumatic brain injury
PT	Polytrauma
iTBI	Isolated traumatic brain injury
AIS	Abbreviated injury scale
ISS	Injury severity score
LOS	Length of stay
IQR	Interquartile range
TRA	Traffic-related accident
HR	Hazard ratio
CI	Confidence interval
